# Current Knowledge on River Buffalo Meat: A Critical Analysis

**DOI:** 10.3390/ani11072111

**Published:** 2021-07-15

**Authors:** Liliana Di Stasio, Alberto Brugiapaglia

**Affiliations:** Department of Agricultural, Forest and Food Sciences, University of Torino, Largo Braccini 2, 10095 Grugliasco, Italy; alberto.brugiapaglia@unito.it

**Keywords:** river buffalo, meat, production, quality

## Abstract

**Simple Summary:**

Buffaloes are reared for different purposes, primarily for milk and dairy products. Meat is often a secondary product and mainly derives from old animals at the end of their productive or working life. However, in recent years buffalo meat has gained increased popularity due to its nutritional properties. Therefore, a huge economic potential might arise from the development of the meat sector in buffalo breeding. This review provides an overview of the recent advances in the knowledge on river buffalo meat, with a special focus on quality traits, and offers insights for future research aimed at improving the meat sector in this species.

**Abstract:**

The estimated world population of water buffalo counts around 204 million head, mostly reared for milk production. However, buffaloes also largely contribute to the meat sector, with around 4.3 million tonnes produced in 2019, mainly derived from old animals at the end of their productive or working life and only to a small extent from young animals. Therefore, buffalo meat production has been generally considered unsatisfactory for both quantity and quality. In fact, the dressing percentage is generally lower than 50% and the meat is considered of poor quality mainly due to its dark colour and reduced tenderness. However, in recent years, the healthy properties highlighted by some studies have led to a renewed interest in buffalo meat, with a parallel increase in research. Therefore, this review aims at providing an updated picture on carcass and meat quality traits in river buffalo, with special attention to the intrinsic and extrinsic factors contributing to their variability. The research done so far has demonstrated that river buffaloes can efficiently contribute to the quanti-qualitative production of meat, provided that the meat supply chain is specifically organised for this purpose. The analysis of the available data also showed that further research is needed on the factors affecting meat production in order to gain greater knowledge essential for planning more targeted interventions.

## 1. Introduction

According to the FAO database [1], in 2019 the world buffalo population accounted for 204,342,419 head, with a continuous increasing trend over the last decades (+127.5% since 1961 and +24% since 2000). The large majority of buffaloes (97%) are reared in Asia, mainly in India (109.8 million), Pakistan (40.0 million), and China (27.3 million). In other regions, buffaloes are generally concentrated in single countries: Egypt in Africa (3.5 million), Brazil in the Americas (1.4 million), and Italy in Europe (0.4 million). Such a wide world distribution implies the existence of a large variety of situations, with the herd size varying from a few to hundreds of animals, which in turn results in very different breeding practices (for a detailed description, see Borghese and Mazzi [2]). Limited to river buffalo, 22 breeds have been reported [3], with a great variation in morphological and productive traits.

Buffaloes are reared for different purposes, primarily for milk and dairy products. Draught power also remains of prime importance, especially for smallholders in many areas, such as in most southeast Asian countries [2]. Buffaloes also largely contribute to meat production, which mainly derives from old animals at the end of their productive or working life and only to a small extent from young animals.

The world buffalo meat production recorded in 2019 (Table 1) was 4,290,212 tonnes, from 27,692,388 slaughtered head, of which more than 90% is attributable to Asia [1]. As expected, within Asia, the main contributing countries were India (42%), Pakistan (28%), and China (17%). In the other areas, the meat production was concentrated in the single countries where buffaloes are reared.

The different breeds of origin and the different age at slaughter result in a great variation in carcass and meat traits. In addition, the general lack of breeding systems specific for meat production and proper pre- and post-slaughter technologies lead to poor quantitative and qualitative performances that often do not meet the expectations of either producers or consumers [4,5]. On the other hand, buffaloes exhibit positive characteristics as well. They are superior to cattle in exploiting the low-quality feeds typical of many rearing areas and demonstrate a great capacity for adaptation to a wide variety of management conditions [6,7].

Moreover, in recent years buffalo meat has gained increasing popularity due to beneficial properties highlighted by some studies, so much so that it has been defined as “the healthiest meat among red meats for human consumption,” mostly thanks to its reduced fat and cholesterol content [8]. In Australia, the TenderBuff^®^ brand was recently developed to promote the consumption of buffalo meat, offering a quality-assured product with a grid-pricing system based on the specification a carcass achieves [9]. A trademark to valorise buffalo meat was created in Italy, too, called *Sapore di Campania* (“taste of Campania”), from the name for the Campania region (South Italy), where buffalo farming is concentrated. To be placed on the market with this brand, the meat must meet quality requirements for the content of fat, protein, cholesterol, and iron [10].

All the above considerations highlight the huge economic potential that might arise from the development of the meat sector in buffalo breeding, especially in the main producing countries, that is, India, Pakistan, China, and Egypt, which cover around 95% of global buffalo meat production, but where this potential is not efficiently exploited. For these reasons, in the last few years a considerable amount of work has been done that focuses on buffalo meat and factors that can influence its properties, as documented by several authors [4,8,11,12,13,14].

The aim of this review is to provide an overview of the current knowledge on river buffalo meat, with special focus on quality traits, and to offer insights for future research aimed at improving the meat sector.

## 2. Growth and Carcass Quality

When analysing and comparing the published data on buffalo production, caution must be taken because the animals under investigation in the different studies were very heterogeneous with respect to several variables, including breed, sex, age, diet, and slaughter weight. The wide geographical, pedological, and climatic differences among the rearing areas all over the world, with their great impact on the breeding systems, were further sources of variation in the meat performances.

According to the published literature, the carcass yield in river buffalo varies between approximately 45% and 59% (Table 2), although about 2/3 of the values are below 50%. This is generally low compared to cattle, mainly due to the significantly higher proportion of head and hide in buffalo [15,16,17,18]. Even lower dressing percentages (about 47%) were obtained from buffalo cows at the end of their reproductive life or culled for reproductive problems, and again the performances were lower than those of bovine females [19]. As can be inferred from the published papers, breed is an important factor affecting carcass yield, but few data exist on the comparison of different breeds [20].

As the age increased, slaughter and carcass weights also increased, but not dressing percentage, which tended to decrease [29,30,31,37]. However, higher slaughter weight corresponded to higher meat yield, due to the lower incidence of bones [20,29,37]. Carcass quality was affected by gender, with a significantly higher dressing percentage in males compared to females, independent of the breed [20]. Moreover, a higher proportion of muscle (67.5% vs. 62.5%) and a lower proportion of fat (12.5% vs. 19.5%) was reported for males compared to females, whereas the proportion of bone was not affected [38]. The effect of castration is controversial, showing either higher yields, as reported for castrated Mediterranean buffaloes due to the lower incidence of skin [34], or the absence of differences, as observed in buffaloes of the “Brazilian type” [31].

The large influence of the farming system on carcass traits has been reported by several studies, with diet playing a major role. In fact, the limited weight gain and carcass yield in buffalo depend in part on the very poor pasture available in most of the rearing areas, and in part on the scarcity of knowledge on the nutritional requirements specific for animals intended for meat production [39], combined with the difficulties of the local farmers to meet the recommended requirements [40] with the available feed. In fact, when subjected to the same feeding regime, similar performances were obtained for buffalo and cattle [17].

Compared to roughage diets, finishing diets supplemented with protein and energy resulted in higher yields [15,22,32], but independent of the type of components or level [28,33]. These findings seem to indicate that high levels of concentrates are not necessary, as confirmed by results from Nili-Ravi young heifers, where high-level diets (1.5% of body weight vs. 0.5% and 1%) only slightly improved daily gain, due to the better feed efficiency of the animals fed low-concentrate levels [24]. Interestingly, it was also found that a high-energy diet (0.9 milk forage unit/kg dry matter) allows for better growing performance if given early in life compared to later administration, as the buffaloes did not show a compensative weight gain [25]. The replacement of corn grains with sugar beet pulp (15 or 30%) improved daily gain in the Nili-Ravi breed while increasing feed efficiency, with significantly lower costs per kg of weight gain [21]. Feeding costs could be contained by replacing expensive protein sources, like soybean, with other more affordable feeds, such as coconut cakes [41], faba bean [27], or cassava residue [23], without negatively affecting the average daily gain or carcass traits. These studies demonstrate that adequate feeding strategies can help reduce feeding costs while maintaining good performance.

Little information exists on river buffalo about the effect of the housing system, which is known to exert an important role on meat production in cattle [42]. As expected, the few available data on buffalo indicate that proper housing conditions can help improve the meat performance, favouring animal welfare, with positive effects on daily gain. For example, in Surti buffalo calves, the winter use of paddy straw as bedding material either on concrete or a soil surface allowed for the best average daily gain and better economic returns for farmers [43].

From the overall data, it can be concluded that the best meat performance, comparable to that of several cattle breeds [44], can be obtained from young males (approximately 20–36 months old) fed a diet including a period with supplementation of protein and energy, possibly provided using local by-products. However, due to the wide variety of management systems, further research taking into account the multiple aspects related to the farming activity will help identify the breeding conditions able to give the best results in the different situations.

## 3. Meat Quality

Similar to carcass characteristics, the quality of river buffalo meat shows a very high level of variability due to the many affecting factors, both intrinsic and extrinsic.

For ease of reference, in this review the different characteristics contributing to meat quality are subdivided into three categories (chemical and nutritional, technological, and sensory properties), although there are many interrelationships between the different aspects of quality.

### 3.1. Chemical Composition and Nutritional Properties

Meat is an important source of nutrients for the human diet. However, meat composition varies across species and breeds due to many factors of genetic and environmental origin. Therefore, the availability of data on the chemical composition of meat, determined in different breeds and taking into account the different sources of variation, is fundamental to better understand its role in the human diet [45].

In general, data obtained on *Longissimus thoracis et lumborum* muscle show that the chemical composition of river buffalo meat (Table 3) is roughly similar to that of bovine meat. The content of total proteins ranges from approximately 19 to 24 g/100 g of meat, with lower values observed in meat from young animals, which is related to the higher water content [29,46,47,48]. In Anatolian buffaloes, differences were observed between groups of different slaughter weights (200 to 350 kg), with higher protein content in the low-weight group [49]. The effect of the muscle is less clear: Some results indicated lower protein content in *Semimembranosus* compared to *Longissimus dorsi* and *Semitendinosus* [28], whereas others did not detect differences [17]. Diet seems not to influence the protein content [22,28,50].

Collagen is the most abundant protein of the connective tissue and plays a fundamental role in determining meat quality [58]. The few data on collagen content show a wide range of variation, with a value of around 7 mg/g being the most common. Great variability can also be observed within a breed: The lowest (4.22 mg/g) and the highest (7.53 mg/g) values were both reported for Mediterranean buffaloes, which differed in age, final weight, and diet [17,22]. The available data indicate that the collagen content increases with age and its solubility decreases [26,59], which can be explained by the increasing amount of non-reducible crosslinks between collagen molecules [60]. Differences also exist between muscles, with a lower collagen content in *Longissimus dorsi* compared to *Biceps femoris* and *Supraspinatus*, independent of age [59]. Moreover, the large range observed can be ascribed to the different production systems, which in bovine breeds has been identified as an important factor of variation [61]. Further investigations should address this component in buffalo species, considering that collagen content and especially its solubility have been known for a long time to be crucial characteristics of the meat for their contribution to the variation in tenderness [62]. Better knowledge on these aspects would be especially important because tenderness is thought to be lower in buffalo compared to cattle, and this represents one of the factors leading to the negative common opinion on buffalo meat. This is also supported by analytical data that show higher collagen content and lower collagen solubility in buffalo compared to several cattle breeds for different purposes, where the collagen amount is mostly lower than 4 mg/g and its solubility is generally in the range of 20–40% [61,63]. However, it should be underlined that few studies have compared buffalo and cattle performance in trials including animals of both species raised under the same conditions. Therefore, the literature data could differ due to the different experimental conditions more rather than due to real differences between the two species. For example, a comparison between young males (10 months old) of Mediterranean buffalo and Italian Simmental breeds subjected to the same feeding protocol showed a similar proximate composition of the meat, but a significantly higher tenderness in buffalo [17]. In addition, the amount of collagen also has an impact on the nutritional value of meat. In fact, it is the major protein of the connective tissue, which has a lower content of essential amino acids compared to contractile tissue. Therefore, meat with a high percentage of collagen will also have relatively lower nutritive value [64].

Despite the importance of the amino acid (AA) composition from the nutritional point of view, very few data exist on river buffalo meat regarding the content of single AA (Table 4), probably due to the complexity of determination, and this limitation also applies to more investigated species, such as cattle [65].

As reported in Table 4, glutamic acid is the most abundant, followed by aspartic acid and lysine, whereas threonine and methionine are the least represented. AA content has been shown to be affected by age [48,66], with somewhat different results. Other factors that influence the AA content are sex, interaction with the seasons (green or dry), and muscle [59,66]. Therefore, the discrepancies in the effect of age and the large range observed for some AAs can be due to differences in the animals and muscles used for the analysis.

Considering the paucity of data, future research on AA composition of buffalo meat is highly desirable, especially due to its impact on the nutritional and sensory properties of the product. As for the nutritional aspect, the protein efficiency ratio, determined using the rat bioassay as a measure of protein quality, showed the highest value for buffalo meat in a comparison including casein, soy protein isolate, and tempeh [67]. In addition, the large amount of glutamate, defined as “nutritionally” non-essential, but “dietary” essential [68], gives the buffalo meat an important role in human nutrition. In addition, buffalo meat provides moderate amounts of carnosine and anserine, two dipeptides with antioxidant properties [69]. However, linked to the possible role of AAs, other aspects need to be investigated to fully evaluate the nutritional value of buffalo meat. Among these, ageing and cooking methods can be mentioned, considering that significant effects on AA composition were observed in other species [70,71,72].

Another interesting area to investigate is linked to the known contribution of amino acids to the taste of different foods, being precursors of volatile compounds associated with aroma [73,74]. The involvement of AAs in taste was also confirmed for beef [75,76], and in the last few years the metabolomic approach has offered a new tool for the identification of potential biomarkers linked to meat quality traits [77], including taste [78]. Therefore, similar studies in buffalo would help to better understand the physiological mechanisms underlying the differences in taste (but also in other quality traits) observed in relation to different factors, such as breed, sex, ageing, processing, and so on.

Buffalo meat is widely recognised to be poor in fat, which is confirmed by literature reports, with values varying approximately between 1 and 4 g/100 g of meat (Table 3). Thanks to its low fat content, it is commonly believed that buffalo meat is healthier than beef. However, when making comparisons, too-high values are sometimes reported as representative of beef [4,79], but they can be applied only to specific cattle breeds, such as those reared in countries where a high fat content is preferred [80]. Otherwise, the fat in buffalo meat is comparable to that of several dairy or beef bovine breeds, where values varying between 1.06% in the Asturiana breed and 3.21% in the Avilena breed are reported [45]. The situation may even be reversed in favour of beef if buffalo is compared to hypertrophied cattle breeds, characterised by very low amounts of fat [81,82,83]. Therefore, it can be concluded that buffalo meat is really low in fat, but its superiority over beef is not as significant as claimed, or, better to say, it depends on the cattle breed used for comparison.

Age and gender are important factors affecting this nutrient: Fat content increases with age [29,46,48,57] and reaches higher values in females [26,47] and steers [57]. Similarly, it was observed that the fat content increases with increasing slaughter weight [49]. Differences in fat content were observed between muscles, with the *Semitendinosus* showing the lowest value [28,51]. As for feeding, a diet including soybean induced a fat increase, compared to a diet with faba bean [51]. The same occurred with diets supplemented with coconut or palm oil compared to a corn diet [50], or with maize silage compared to alfalfa hay [22]. However, the level of maize silage had no effect on the meat fat content [28].

Moderate levels of cholesterol have been reported for buffalo meat (Table 3), with most of the values around 50–60 mg/100 g meat, which is similar to the range reported for different cuts of beef [84] and for different bovine breeds [81]. A large variability can be observed in Table 3, depending not only on the same factors mentioned for the fat content, but also on the different analytical methods used for the determination. The lowest values in the range were found in Mediterranean buffaloes [51], whereas the highest ones were found in heifers of the Murrah breed [56]. Cholesterol content changes with age: The meat of younger animals has significantly lower content compared to the meat of older ones [48]. Males have lower values compared to heifers [56], but show no difference with respect to steers [57]. Contrary to what was observed for fat, diets with maize silage or alfalfa hay did not induce changes in cholesterol content [22], whereas a higher cholesterol content was observed for a diet with soybean compared to faba bean [51]. In any case, the cholesterol content reported for river buffalo meat is quite low and therefore is not expected to have detrimental effects on human health, also given that serum cholesterol level have been demonstrated to be essentially independent of the cholesterol intake [84].

In recent years the fatty acid (FA) profile of buffalo meat has been extensively studied by several authors, after evidence of its effect on human health [85,86,87]. As the data reported in the literature are often expressed in different units, with “g/100 g of total FAs” being the most commonly used, only those expressed in that unit of measurement are included in Table 5. The other available data were not disregarded, but considered when useful for specific comments.

The data in Table 5 show a great variability in the content of the individual FAs, as well as in the aggregate indices, due to the effects already mentioned for other characteristics. On the whole, the reported values are in the range observed for several European cattle breeds [90]. Similar to beef, the most abundant FAs are oleic (C18:1 n9), stearic (C18:0), palmitic (C16:0), and linoleic (C18:2 n6) acids. Focusing on the aggregate indices, the percentage of SFA, which is known to have adverse health effects [87], is between approximately 35 and 62%, with the highest values observed in the Murrah breed, which also has the lowest percentage of PUFA [56]. However, rather than simply looking at the single FAs, it is more informative to consider some indices, such as the PUFA/SFA and n-6/n-3 ratios, or, even better, the atherogenic (AI) and thrombogenic (TI) indices, which better characterise the health properties of a product [91]. PUFA/SFA ratio values higher than 0.4 are recommended, whereas the n-6/n-3 ratio should not exceed 4.0 [92], but the majority of the data reported for buffalo meat do not respect these limits, as is the case for most bovine breeds [90]. On the other hand, the AI and TI indices, which synthesize the effect that single FAs can have on increasing the risk of coronary heart diseases, are generally lower in comparison to the values reported for several foods of animal origin, including beef (AI: 0.70–0.72, TI: 0.79–1.39), lamb (AI: 1.00, TI: 1.33–1.58), and dairy products (AI: 2.03, TI: 2.07) [93].

As known in cattle [87], the FA profile can be modified by feeding strategies, with the aim of increasing the content of beneficial n-3 PUFA and CLA and reducing SFA. Compared to maize silage, hay induced a lower fat deposition in river buffalo meat, with a higher PUFA/SFA ratio, which is more favourable in relation to human health [22]. The inclusion of soybean oil in the diet, compared to soybean grain, led to higher CLA concentrations and lower SFA contents [94]. The protein source in the concentrate affected only the SFA content, with lower values obtained for diets including faba bean instead of soybean [51]. The energy content of the diet influenced the unsaturated fatty acid composition: MUFA increased with diet with a high energy level, whereas PUFA increased with a low energy level [95]. When subjected to grass feeding, the amount of CLA was higher in buffalo than in cattle, but in any case too low to be considered a significant source for human diets [89].

Meat from young buffaloes (12–18 months) compared to that from adult buffaloes (>3 years) showed superior nutritional quality, due to significantly lower SFA and higher PUFA content [48]. If confirmed, this would be a reason for not delaying the slaughter age beyond two years. Giuffrida de Mendoza et al. [57] investigated the effect of the species (buffalo and cattle) and gender (bulls and steers) on the FA profile at two different ages (19 and 24 months). The results showed that significant differences existed between species and gender, but in general, as the age increased, the differences between buffalo and cattle decreased, whereas those between bulls and steers increased. In the older animals, the PUFA/SFA ratio was higher in bulls relative to steers, combined with a lower atherogenic index, suggesting that meat from intact males has nutritional advantages with respect to steer meat. The meat from males also showed a better FA profile compared to females, thanks to its higher n-3 content, with a consequent lower n-6/n-3 ratio [88]. A study on the impact of buffalo meat consumption on cardiovascular risk revealed possible associations with several beneficial effects, including lower levels of total cholesterol and more favourable vascular parameters [96]. Muscle is a further cause of variability; in fact, when muscles other than *Longissimus dorsi* were considered, significant differences were observed for almost all the FAs, as well as for the FA classes and quality indices, with the *Semitendinosus* showing the most healthy FA profile [51].

In bovine breeds, the FA profile has been shown to also have an influence on the sensory characteristics of the meat [83]. For example, palmitic acid had a negative effect on beef flavour [97], whereas oleic acid has been reported to have a role in good meat flavour and savoury taste [98]. As the FA profile can be, at least to a certain extent, modified by dietary intervention, investigation into this aspect could be of interest in buffalo too.

The importance of minerals in human nutrition is well established, but few investigations have focused on the content of these components in buffalo meat. No differences were observed between sexes, except for iron, with a higher content in males compared to females [99]. Age showed an influence on the content of several minerals, but without a common trend [99,100].

In general, the reported values are similar to those observed for cattle breeds [101] and confirm that potassium, phosphorus, sodium, and magnesium are the most abundant minerals (Table 6). However, of primary importance is the role of meat as a relevant source of essential minerals, particularly iron and zinc. The literature data agree that meat from buffalo is richer in iron compared to the amount (1.0–2.0 mg/100 g) reported for different cuts of beef [102,103], which makes it more valuable from the nutritional point of view. As is well known, iron is fundamental for many bodily functions and its deficiency leads to anaemia, which has been defined as “a public health problem, affecting both developing and developed countries with major consequences for human health as well as social and economic development” [104]. Iron deficiency can be overcome primarily by the diet, where meat plays a fundamental role, supplying a considerable amount of iron mostly in the heme form, which is highly bioavailable, compared to the non-heme iron provided by vegetables [105]. Therefore, diets including buffalo meat could be recommended in case of nutritional iron deficiency. In addition, meat is an important source of zinc, which is the component of hundreds of enzymes involved in cell division and growth. An in vitro model showed that zinc deficiency was responsible for decreased neuronal viability and increased rates of apoptosis, which were reversed by zinc supplementation [106]. Meat is also one of the main food groups providing selenium [107], a microelement that has been suggested to play a central role in human health due to several beneficial properties, including antioxidant effects and reduced risk of cancer and cardiovascular diseases [108]. The selenium content in buffalo meat is comparable to that reported for beef [101] and could be increased by dietary strategies [109,110] to produce Se-enriched functional meat. Studies on mineral content in buffalo meat, apart from its importance in human nutrition, would be important to clarify its role in animal performance and beef quality traits, as demonstrated in bovine breeds [109,111].

Meat is rich in vitamins, which are essential nutrients involved in biological functions. In particular, meat and other animal-derived foods are the only source of vitamin B12, the deficiency of which results in haematological and neurological disorders. Despite the interest in how it relates to human health, vitamin content in buffalo meat has been poorly investigated [112], and research would be desirable to fill this knowledge gap.

### 3.2. Technological Properties

The technological traits of meat, which are the main determinants for consumers’ appreciation, are strictly interrelated. Therefore, dealing with them separately, as in this review, is quite a reductive approach. To compensate for this limit, references will be made to specific reviews in order to give insight into more basic aspects.

In Table 7 the range of values obtained on *Longissimus thoracis et lumborum* muscle for the traits that determine the technological quality of river buffalo meat is reported. Compared to those covered in the previous paragraphs, the traits considered here show additional variability, depending not only on the pre- and post-mortem factors, but also on the time elapsed since slaughter.

The pH, but especially its post-mortem decline, is a very important trait, because it reflects the effects of the pre- and post-slaughter procedures and, in turn, influences several meat characteristics, such as colour, water holding capacity, tenderness, juiciness, and flavour [117]. Therefore, pH has a great impact on meat quality. Of the vast amount of published data on pH in buffalo meat, only those specifying the moment of the measurement were considered, and, among the remaining, only those determined at 24 h post-mortem were retained. Overall, most of the values reported for the pH of buffalo meat (Table 7) are within the range typical for animals subjected to proper handling, a necessary condition for preventing possible negative effects on the meat quality. However, it is likely that this is not the general situation of retail meat, because the data in the literature mostly refer to animals reared and managed in experimental conditions, which often differ from the usual production systems.

The influence of age on pH was investigated, but with controversial results, indicating either higher values in younger animals [116], or in the older ones [48], or an absence of any effect [114]. The large variability in the age of the animals used in the different studies, together with the differences in the age range within studies, might explain the conflicting results. Sex also seems to affect pH, with higher values reported for males [88,118]. No effects of the diet on pH were reported [33,50]. The pH decline after slaughter was significantly slower in crossbred buffalo meat compared to crossbred cattle beef, with ultimate pH reached in buffalo at 48 h post-mortem [119]. The same trend can be seen from the data of Spanghero et al. [17] in Mediterranean buffalo. The pH continued to decline until day 2 in young buffaloes and until day 6 in the old ones, when a value of 5.42 was reached [115].

In addition, the temperature to which the carcasses are subjected has a great effect on pH [59], making it of great importance to identify the best pH/temperature profile in order to assure satisfactory meat quality [120]. This aspect has been poorly investigated in buffalo and deserves more attention, considering that in some countries, carcasses are often rapidly chilled, and this procedure can cause cold shortening, with the consequent worsening of meat tenderness and colour. Studies in river buffalo demonstrated that electric stimulation could be used to prevent the negative effects of fast cooling [121], eventually combined with hip suspension [122]. Despite its practical importance for the relationship with cold shortening and other meat quality traits [123], only a few authors [26,35] have analysed sarcomere length in buffalo meat, showing higher values in young animals (1.83 µm in 18 months old animals vs. 1.51–1.56 µm in animals over the age of 10 years), considered indicative of more tender meat [26].

As far as we know, no studies in buffalo have considered the effect of stress, which in other species has a well-known impact on meat pH, and consequently on the many quality traits related to pH. Stress can be caused by a wide range of environmental factors, including the production system, duration of transport to the slaughterhouse, and processing procedures at slaughter plants [124]. Therefore, investigations on these aspects would be desirable in the buffalo species as well in order to define the optimal conditions for the meat supply chain to assure a high-quality product.

The water-holding capacity (WHC) is the ability of meat to hold moisture, the largest part of which is represented by water associated with muscle proteins, and it is highly influenced by the rate and extent of pH decrease [117]. In addition to pH, many other factors can affect WHC, including sex, muscle type, and storage temperature. In turn, WHC influences different meat characteristics, such as colour and eating quality [125]. Therefore, this trait is very important both for producers and consumers.

As several different procedures were used to measure the trait, Table 7 reports only the results obtained with the two reference methods: drip loss in raw meat and water loss in cooked meat [126]. Results obtained with other methods are considered when general information can be deduced. Looking at the literature data, WHC is one of the quality traits for which a very high heterogeneity of the results can be observed when causes of variation are studied. For example, the results on the trend of WHC related to meat ageing show either a decrease in cooking loss [88] or an increase, but depending on the muscle [127]. The effect of sex is also unclear, with data indicating better WHC in males [26], or no differences between sexes [88]. Conflicting results were also obtained when the effect of the diet was investigated, because some studies did not evidence any influence [50,53], whereas significant differences in cooking loss were reported for different finishing diets (alfalfa hay or maize silage) and correlated to different fat content [22]. WHC seems to not be affected by age [26,29,48].

Colour is the principal meat characteristic that drives consumers’ purchasing decision. The preferred colour of fresh meat is bright red, and it is exactly the darker colour usually displayed by buffalo meat compared to beef [15,17,128] that contributes to the negative perception of its quality, because darker meat is often associated with a less fresh product, or as being derived from old animals, and therefore of lower eating quality. It has been demonstrated that the darker colour of buffalo meat depends on the myoglobin content (0.393 ± 0.005 g/100 g of tissue) and consequently on metmyoglobin, which are almost twice as high as bovine meat, more so than on its oxidation rate [129].

Meat colour can be measured using a reflectometry-based instrument, which generates CIEL*a*b* values (L*: lightness, a*: redness, b*: yellowness) from which the parameters Chroma (C*), related to the intensity of colour, and hue angle (h*), related to the change in colour from red to yellow, can be calculated. Of the many data available on colour parameters, only those measured after blooming were included in Table 7. Many pre- and post-mortem factors affect meat colour [130]. Higher L* and b* and lower a* were reported for young buffalo [48], which could be explained by the lower total pigment in meat from young compared to old animals [26,114]. The comparison of meat from animals differing in slaughter weight (200 to 350 kg) detected significant differences between colour parameters measured at 1 h, but very similar values measured at 24 h [49]. Significant differences exist between muscles, with *Longissimus dorsi* characterised by lower lightness [17,28] and higher redness [17] compared to *Semitendinosus*. As for the effect of gender, lower L* and higher a* values in females [88] and higher L* and b* in castrates [131] were reported compared to males.

Contrasting results were obtained when the influence of the feeding system was considered. Several authors did not find any effect of diet on the colour parameters [28,41,53]. Conversely, the inclusion of ryegrass was shown to determine a worsening of the colour parameters measured at 5 days of ageing, which, however, reached values similar to the control in more prolonged ageing periods [132]. Significantly higher a* and b*, as well as higher oxymyoglobin content, were observed in the meat of animals fed maize silage compared to alfalfa hay [22]. Diets supplemented with vitamin E (600 UI) affected colour parameters according to the muscle: In the *Longissimus dorsi* and *Semimembranosus* muscles redness increased, whereas in *Semitendinosus* redness and yellowness decreased [133].

In addition to differences in the category of the slaughtered animals and the production systems, other factors, including mainly pre-slaughter handling of the animals, post-slaughter treatment of the carcass, and length of the storage period, can affect the meat colour [134]. Sen et al. [135] observed that the bloom time had a significant effect on all the colour parameters, with values continuously changing for 60 min, leading to a significant decrease in L* and increase in a* and b*. The same authors showed that the retail display time also had a significant effect on instrumental colour characteristics of buffalo meat, with the a* value significantly increasing after 12 h of display. As a result, the meat became darker immediately after exposure to air, and the change is explained by the fact that over time meat loses its ability to reduce metmyoglobin to oxymyoglobin, as confirmed by Kiran et al. [115], who observed a decrease of myoglobin parallel to the increase of metmyoglobin from day 0 to day 6.

The effect of ageing for more prolonged periods on the colour of buffalo meat has been analysed by several studies, which, however, were very heterogeneous in the material used (age of the animals, treatment of the carcasses, sample preparation and storage, measurement methods) and the ageing period considered (days, weeks, months). In meat samples vacuum packaged and aged at refrigerated temperature, little effect on colour was seen between day 7 and 14, even though the difference in yellowness was significant, with an increase at day 14 [28]. Instead, Ekiz et al. [88], in samples subjected to similar conditions for ageing, observed an increase in L* and a*. A weekly analysis of the colour parameters from day 0 to day 21 showed an increase in L* in buffaloes slaughtered at 20–24 months, and an increase in b* in heifers 32–36 months old. As a general indication, these studies indicate that during refrigerated ageing, lightness increases, whereas for a* and b* it is more difficult to find a common trend. The use of vitamin C [135], eventually combined with lactic acid and clove oil [136], proved to be useful for improving meat colour stability.

Further studies on the factors affecting meat colour would be necessary to provide few and simple practical indications on how to improve this important characteristic according to the local situations. In fact, in this respect a large variability exists in the procedures implemented in the different countries before buffalo meat is displayed at retail, with two extreme situations: On one side, there are countries where, after slaughtering, the carcass is subjected to an ageing period of some days in refrigerated conditions before it is sold; on the opposite side, there are countries where meat is displayed at room temperature in the retail outlets immediately after slaughter and sold throughout the day.

If meat colour guides consumers in their purchasing decision, tenderness is the quality trait consumers appreciate most. As seen for other technological properties, tenderness has long been known to depend on many intrinsic and extrinsic factors [137], whose effects have been, and continue to be, deeply investigated to identify interventions able to improve this characteristic, in order to satisfy the consumers’ expectations.

Warner–Bratzler shear force (WBsf), which is a measure of the force required for shearing a sample of cooked meat, is the most widely used instrumental method to determine tenderness. For river buffalo meat, the values of WBsf, measured at day 7 post mortem, show a wide range of variation (Table 7). However, a comparison of the absolute values reported by the different studies is almost impossible, because not only the samples used but also many steps of the analysis can affect the results, even though standardised protocols have been developed [138]. Therefore, once again, the main attempt of this review is to provide general information on the affecting factors and their effect. Another aspect to underline is that the large majority of the WBfs measurements were taken on the *Longissimus thoracis et lumborum* muscle, which is the reference muscle for meat-quality analyses. For this reason, Table 7 is based only on the values reported for this muscle. However, the few studies that also considered other muscles highlighted significantly higher WBsf values in comparison to *Longissimus thoracis et lumborum* [17,28,132].

Meat from young animals shows lower WBsf values compared to meat from older ones [26,48,59,115,116], which is not unexpected considering the significant increase in collagen content and decrease in its solubility at older ages, together with lower sarcomere length in older animals [26]. On this basis, the fact that a large part of the world’s buffalo meat derives from spent animals certainly contributes to the negative perception that buffalo provides a less tender meat in comparison to cattle. Concerning gender, some authors reported lower WBsf values for females, independent of the age class and storage length [113], as well as lower WBsf for castrates compared to entire males [131]. On the contrary, other authors [88] found no differences between sexes in younger animals of the same age. No significant effects of the diet were reported [22,50,53,132]. As previously discussed, the procedures in the abattoir can have a strong impact on pH and WHC, and consequently on WBsf.

Ageing is confirmed as the most important factor that influences the meat tenderness, independent of other factors, including sex, age, and breed. In fact, decreasing values of WBsf from day 0 to various ageing periods were consistently observed [56,88,113,115,119], with a parallel increase in the myofibrillar fragmentation index (MFI), especially evident in younger animals [56,115] and in agreement with observation that the post-mortem tenderization depends mainly on the degradation of the myofibrillar proteins due to protease activity [139]. The evolution of WBsf during ageing was shown to be different according to the animal category: In heifers 32–36 months old the WBsf value decreased until day 7 and then remained almost constant, whereas in males 20–24 months old WBsf continued to decrease until day 21 [56]. Such studies on the WBsf trend in the different categories have great practical importance, because they make it possible to identify the best ageing protocol to supply buffalo meat of better quality.

As mentioned above, in some countries it is customary to sell buffalo meat on the same day of slaughter, a practice that prevents meat from reaching the desirable tenderness, considering that between 12 to 24 h post-mortem the maximum toughness is observed [139]. Therefore, this is an additional reason that can contribute to the idea that buffalo meat is of lower quality. Actually, when buffalo meat is derived from young animals and is subjected to proper ageing, it shows WBf values even lower than beef [15,17,119], possibly due to the higher protease activity [140]. Treatment with plant proteases such as Cucumis or ginger extracts [55] or the use of ammonium hydroxide as a marinating agent [141] was suggested to tenderise tough meat provided by spent animals, thereby increasing its economic value. The preparation of processed products especially from minced meat, including patties, sausages, and nuggets, is another way to add value to meat of lower quality [8,142].

### 3.3. Sensory Properties

The sensory quality of a product is a set of characteristics perceived by the senses, and it is the main determinant of its acceptance by the consumers. Based on human perceptions, sensory analysis provides information not provided by instrumental analyses. Many factors—mainly related to the meat sample under analysis, the panel of assessors, and the conditions under which the test is carried out—can affect the results, making sensory analysis a very complex subject. If interested in more details on the principles and methods of meat sensory evaluation, readers can refer to [143,144,145].

In Table 8, the range of evaluation scores obtained by trained panellists for different sensory traits of fresh buffalo meat is reported. As the available studies differ in the scale used for evaluation, only results expressed in the same scale are shown for each trait. Apart from that, the comparison of the results remains difficult due to the wide variety of materials and methods used. Therefore, attention will be focused on inferring general information more than discussing single values.

As shown by the range of variation of the considered attributes, the sensory quality of buffalo meat is quite variable, but this is not surprising on the basis of the many affecting factors mentioned above. For most of the traits, the minimum values are quite low, indicating poor perceived quality, but on the other hand, the highest scores in the range are often close to the best extreme of the scale, thus proving that buffalo meat can reach a satisfactory organoleptic quality, similar if not superior to that of beef. For example, buffalo meat evaluated by a consumer panel obtained a higher score for tenderness compared to that of Angus breed reared in the same conditions [146]. When compared to the Simmental breed, buffalo meat was rated by consumers as better in flavour, and this resulted in a higher overall liking, but only when cooked [17]. The higher tenderness observed in buffalo meat compared to beef from Brahman cattle of the same age, gender, and diet was explained by the significantly higher calpain activity found in buffalo [147]. Similar results were obtained when trained panellists were used. A study carried out in the Philippines—where the animals are generally slaughtered at the end of their productive life, thereby providing meat of very poor quality—showed that meat of young crossbred buffalo (18–24 months old) was comparable or even slightly superior to that of crossbred cattle in sensory score for colour, tenderness, and flavour [15]. On the other hand, when comparing meat obtained from local stores, beef was superior to buffalo meat in appearance, tenderness, and juiciness [129]. Taken together, these data are further evidence that results obtained in experimental trials do not necessarily reflect everyday situations.

Few studies investigated the extrinsic factors possibly affecting the sensory properties of buffalo meat, most likely because of the complex nature of the analysis. The comparison of meat from different age groups (6 months to over 4 years) showed a significant decrease in tenderness, with a parallel increase in flavour; interestingly, juiciness and overall acceptability improved up to the age of 4 years and then decreased in animals above that age [113]. No differences were observed between meat from males or females 2–2.5 years old [88], whereas higher tenderness was reported for spent males compared to spent females [26]. The effect of the diet is less clear. No differences in sensory quality were detected by trained panellists between buffaloes supplemented with agro-industrial by-products or with the traditional corn and soy [50], as well as between animals finished in the traditional or silvopastoral system in Brazil [53]. Supplementation with a low dose (600 UI/day) of vitamin E in the diet positively improved the tenderness and juiciness of the meat, but it had a negative effect on colour uniformity [148].

Apart from the previously mentioned factors, the evaluation of the sensory properties of a product is highly influenced by the method of analysis, and in this regard the characteristics of the assessors play a major role. In fact, the consumers’ evaluation can be affected not only by variables that can be easily taken into account, such as sex and age, but also by psychological emotions, which are much more difficult to analyse [145]. An investigation into the relationships between the sociodemographic profile of the consumers and the sensory evaluation of buffalo meat and beef [146] revealed that the gender of the assessors did not influence any of the sensory traits, whereas the age affected odour and colour, with people over 50 giving higher scores. However, the main determinant for four out of the five sensory traits was income, with low-income people giving the highest scores. Another interesting study carried out in Brazil on consumers’ attitude towards buffalo meat highlighted that it was appreciated especially by young people, who stated that information about the product characteristics would favour their willingness to buy, and that they would also a higher price if presented with a certification of quality [149]. This kind of analysis offers interesting insights into the interpretation of the results of sensory analysis and can suggest possible marketing strategies.

The overall findings point to the need for further research in order to reach a better understanding of the factors that can influence meat sensory quality, which is the basic step for implementing an increasingly high-quality meat production chain, through action on the determining factors that are most easily manageable.

## 4. Conclusions

There is scientific evidence that buffaloes can efficiently contribute to the quanti-qualitative production of meat, provided that the meat supply chain is specifically organised for this purpose. In fact, an analysis of the currently available data shows that yield and quality of river buffalo meat is satisfactory and comparable to that of cattle, as long as the buffaloes are managed and fed as meat animals, slaughtered at an appropriate age and subjected to proper pre- and post-slaughter operations. Although not very extensive, research done so far allows us to identify the principal factors that should be considered for improving some of the meat traits in the buffalo species. However, many aspects remain to be investigated in order to gain wider knowledge for planning targeted interventions.

The major issue with providing indications on how to improve buffalo meat is that buffaloes are reared in very different areas all around the world, some of which rely on advanced production systems, whereas others, which are the large majority, have to face adverse environmental and socio-economic conditions that prevent them from moving to more effective management. Some valuable practical indications can be drawn from the analysed studies, demonstrating that a gradual shift to more efficient management, including farming system, slaughter operations, and meat distribution, is possible and could offer the chance for a progressive improvement in the buffalo meat chain in less favourable situations as well. Even though not covered by this review, it is essential to point out that genetic programmes are a necessary complement to the activities implemented for the improvement of meat production in the buffalo species. In addition, for best results, any measures taken should be accompanied by an appropriate marketing policy to promote the sector. To achieve these goals, the involvement of local bodies at both the technical and political level is highly desirable. Of course, financial efforts are necessary to support these activities, but an economic return in the medium term can be expected.

## Figures and Tables

**Table 1:** Buffalo meat production [1].

Area	Head	Tonnes
World	27,692,388	4,290,212
Africa	1,088,548	365,588
Americas	29,701	6720
Asia	26,469,363	3,896,627
Europe	104,776	21,277

**Table 2:** Growth and carcass traits of river buffalo.

Trait	Range	References
Average daily gain, ADG (kg)	0.50–1.13	[21,22,23,24,25]
Age at slaughter (yrs)	>1–<10	[25,26,27,28,29]
Weight at slaughter (kg)	223–540	[28,29,30,31]
Carcass yield (%)	45–59	[15,16,17,19,20,22,27,28,29,32,33,34,35,36]

**Table 3:** Chemical composition of river buffalo meat.

Trait	Range	References
Moisture (g/100 g)	71.33–77.18	[15,16,17,26,28,29,32,33,35,47,48,50,51,52,53,54,55]
Ash (g/100 g)	0.57–1.82	[15,17,22,28,32,35,46,48,50,51,52,53]
Protein (g/100 g)	19.10–23.87	[15,17,22,26,28,33,35,46,47,48,50,51,52,53,54,55]
Collagen total (mg/g)	4.22–7.53	[17,22,55]
Collagen solubility (%)	7.40–20.90	[26]
Fat (g/100 g)	0.9–3.98	[15,17,22,26,28,32,33,35,46,47,48,50,51,52,53,54]
Cholesterol (mg/100 g)	32.20–123.79	[15,22,48,51,52,54,56,57]

**Table 4:** Amino acid (AA) content (g/100 g of meat) of river buffalo meat.

AA	Range	References
Alanine	0.62–1.10	[48,66]
Arginine	0.71–0.78	[48]
Aspartate	0.89–1.05	[48]
Cysteine + cystine	0.30–0.42	[66]
Glutamate	1.80–2.22	[48]
Glycine	0.53–0.94	[48,66]
Histidine	0.35–0.51	[48]
Isoleucine *	0.50–0.56	[48]
Leucine *	0.87–0.88	[48]
Lysine *	0.84–1.07	[48]
Methionine *	0.16–0.20	[48]
Phenylalanine *	0.42–1.02	[48,49,50,51,52,53,54,55,56,57,58,59,60,61,62,63,64,65,66]
Serine	0.50–0.51	[48]
Threonine *	0.10–0.35	[48]
Tyrosine	0.43–1.00	[48,66]
Valine	0.57–0.67	[48]

* Essential amino acid.

**Table 5:** Fatty acid content (g/100 g FAs), fatty acid classes (% total FAs), and nutritional indices of river buffalo meat.

FA	Range	References
C14:0	0.89–3.63	[17,22,51,56,57,88]
C15:0	0.15–0.56	[51,56,57,88]
C16:0	18.13–27.81	[17,22,47,51,56,88]
C16:1	0.98–3.12	[50,56,57,88]
C17:0	0.28–1.52	[22,47,51,56,88]
C17:1	0.43–0.98	[22,88]
C18:0	19.80–33.01	[17,22,51,56,88]
C18:1 n9	27.80–40.18	[17,22,47,51,56,57]
C18:2 n6 (LA)	2.95–12.46	[17,22,51,57,88]
C18:2 9c11t (CLA)	0.46–1.27	[22,89]
C18:3 n3 (ALA)	0.23–1.38	[17,22,51,56,57,88]
C20:0	0.07–0.32	[22,51,56,57,88]
C20:5 n3	0.07–0.29	[22,51,57,88]
C22:0	0.02–0.24	[22,51,56,57]
C22:6 n3	0.03–0.24	[22,56,57]
C24:0	0.05–1.01	[22,51,57]
SFA	34.87–62.25	[17,22,51,56,57,88]
MUFA	32.80–61.16	[17,22,51,56,57,88]
PUFA	2,48–18.63	[17,22,51,56,57,88]
PUFA/SFA	0.10–0.36	[22,51,57,88]
PUFA-n3	0.58–2.51	[17,22,56,57,88]
PUFA-n6	3.23–17.43	[17,22,57,88]
n-6/n-3	3.82–8.61	[17,22,51,57,88]
Atherogenic Index	0.35–0.60	[51,52,57,88]
Thrombogenic Index	0.65–1.51	[51,52,57,88]

**Table 6:** Mineral content (mg/100 g of meat) of river buffalo meat.

Mineral	Range	References
Ca	3.59–10.31	[99,100]
P	202.53–223.78	[100]
K	312.84–383.98	[99,100]
Na	61.42–72.95	[99,100]
Mg	22.35–25.34	[99,100]
Fe	2.28–2.62	[99,100]
Zn	3.65–4.34	[99,100]
Cu	0.09–0.16	[53,99,100]
Mn	0.01–0.02	[100]
Se	0.017–0.20	[53]

**Table 7:** Technological traits of river buffalo meat.

Trait	Range	References
pH_24_ *	5.29–5.75	[17,49,53,56,113]
Drip loss (%)	1.3–4.18	[22,49]
Cooking loss (%)	25.7–34.3	[17,22,49,50,114]
Lightness, L *	34.15–59.02	[17,28,35,41,53,88,114]
Redness, a *	10.8–26.55	[17,28,35,41,53,88,114]
Yellowness, b *	6.8–18.3	[17,28,35,41,53,88,114]
Myoglobin (mg/g)	2.36–3.59	[35,115,116]
Metmyoglobin (%)	31.60–65.08	[35,115]
Sarcomere length (µm)	1.51–1.83	[26,35,116]
WBsf ** (N)	36.89–93.81	[17,22,26,55,56,116]
WBsf ** (kg)	1.56–5.60	[53,113]

* pH measured at 24 h post-mortem; ** Warner–Bratzler shear force.

**Table 8:** Sensory traits of river buffalo meat.

Trait (Scale)	Range	References
Colour (1–7)	4.76–6.14	[15,32]
Appearance (1–8)	5.77–6.85	[26,55]
Odour (1–8)	4.53–4.54	[88]
Flavour (1–8)	4.79–7.26	[26,55]
Off flavour (1–7)	1.24–1.28	[15,32]
Juiciness (1–8)	4.23–7.00	[26,55,88]
Tenderness (1–8)	4.00–7.02	[26,55,88]
Overall acceptability (1–8)	4.66–7.11	[55,88]

## Data Availability

Not applicable.
